# Methodological approach to optimize a step-by-step deterministic linkage of SNDS data with a clinical database (FREGAT) of gastric/gastroesophageal junction adenocarcinoma in France: Pitfalls and learnings

**DOI:** 10.1371/journal.pone.0333667

**Published:** 2025-11-07

**Authors:** Magali Laborey, Audrey Lajoinie, Jonatan Freilich, Emmanuelle Samalin, Olivier Bouché, Guillaume Piessen, Matthias Stoelzel, Andrew Chilelli

**Affiliations:** 1 RCTs, Lyon, France; 2 Parexel, Stockholm, Sweden; 3 Institut Régional du Cancer de Montpellier, University Montpellier, Montpellier, France; 4 Department of Digestive Oncology, CHU Reims, Reims, France; 5 University Lille, CNRS, Inserm, CHU Lille, UMR9020-U1277–CANTHER–Cancer Heterogeneity, Plasticity and Resistance to Therapies, Lille, France; 6 Department of Digestive and Oncological Surgery, Claude Huriez Hospital, CHU Lille, Lille, France; 7 Astellas Pharma GmbH, Munich, Germany; 8 Astellas Pharma Europe Ltd., Addlestone, United Kingdom; Athens Medical Group, Psychiko Clinic, GREECE

## Abstract

**Purpose:**

Survival rates in the European population with gastric and gastroesophageal junction (G/GEJ) adenocarcinoma remain low. Epidemiologic research is warranted to understand the population size, unmet need, and current treatment patterns of G/GEJ adenocarcinoma. The objective of this research was to develop an algorithm to link patients across the FRench EsoGAstric Tumours (FREGAT) and Système National des Données de Santé (SNDS) databases to develop a real-world dataset for G/GEJ adenocarcinoma.

**Methods:**

A step-by-step, indirect, deterministic record linkage algorithm was developed to match patient records from the FREGAT and SNDS databases. Corresponding variables in each data source were matched at an individual level. Each step in the linkage process used a given scoring criterion; the linkage process proceeded until a unique pair of patient records had equal observations across the databases, at which time patient data were considered linked. Due to the large number of potential matches, the linkage process was performed in two parts: first, matching on the stratified population using individual corresponding variables, and second, by linking without any stratification. Descriptive and inferential statistics were used to assess validity of the linkage process. This study was approved by the National Expertise Committee (Ethical and Scientific Committee for Research, Studies and Evaluations in the Field of Health; 5758940) and the French Personal Data Protection Agency (CNIL; 92 1441/DR 2022 088).

**Results:**

Of 1617 patients included in the FREGAT database, 1385 (85.7%) were successfully linked to the SNDS database. A majority of the linked patients (1159 [83.7%] of 1385) were matched in the first part of the linkage process.

**Conclusion:**

We established an algorithm that enabled linkage of the FREGAT and SNDS databases that may be applied to capture additional data related to G/GEJ adenocarcinoma in France.

## Introduction

Prognoses of gastric and gastroesophageal junction (G/GEJ) adenocarcinoma remain poor. In Europe, the current 5-year survival rate for all stages combined is 20.7% for gastric cancer and 9.2% for esophageal cancer [1]. Low survival rates in the European population are partially due to a continued lack of recognition of early symptoms and diagnosis at advanced stages of disease [2,3]. In comparison, 5-year survival rates for G/GEJ adenocarcinoma are markedly higher in Japan (62.1%, gastric cancer; 31.6%, esophageal cancer) and South Korea (76.5%, gastric cancer; 38.0%, esophageal cancer), where increased awareness of disease burden and routine screening have resulted in increased early-stage tumor detection and treatment, and improved survival [4–6]. Further epidemiologic research is warranted in Europe to better understand G/GEJ adenocarcinoma burden and improve treatment [7].

The linkage of patient records is an important aspect of epidemiologic and public health research that can help clinicians and data analysts better understand chronic and multifactorial diseases. Linking patient records across databases can widen the scope of research and minimize evidence gaps [8]. Research based on linked patient records can drive policy and improve clinical practice [8,9]. However, data extracted from linked patient records may be vulnerable to errors and biases because they require researchers to balance quality and privacy. Developing methods to adjust for these biases is necessary to produce robust data and evidence that can inform policy [10]. The GUidance for Information about Linking Data sets (GUILD) suggests that when linking new databases/registries, it is necessary to allow researchers using linked data to be aware of any potential biases [9,11].

In France, record linkage using the French National Health Data System (Système National des Données de Santé [SNDS]) database, the world’s largest continuous homogenous claims database with data covering approximately 99% of the French population (over 66 million people) from birth (or immigration) to death (or emigration), has been shown to improve the quality of data and can generate new pathways for research in several fields of study [12,13]. The SNDS database is a key resource in linkage studies for France as it is an exhaustive claims database with a high degree of completeness, which can help in retrieving information regarding patients who may have been lost to follow-up, a feared source of bias, and can provide longer follow-up data [14]. Several studies have been conducted using SNDS data linked with data from other registries/databases [13–16]. Combining patient- or event-specific data from linked patient datasets has been shown to improve data quality and reduce any evidence gaps for chronic medical conditions, such as chronic kidney disease, cancer, cardiovascular diseases, and infections, across France [17]. Thus, a linked dataset for G/GEJ adenocarcinoma in the French population provides an opportunity to study the disease in more depth and promotes research to improve outcomes in this underserved patient population better than either of the two databases in isolation.

Linked patient datasets from two large French data repositories, the FRench EsoGAstric Tumours (FREGAT) clinico-biological database, which contains rich clinical cancer data, and the SNDS database, can provide deeper insights on the prevalence, burden, outcomes, and treatment patterns for G/GEJ adenocarcinoma in the French population and clinically defined G/GEJ subgroups. FREGAT is a prospective database of clinical, biological, and tumor data for adult patients treated for esophageal or gastric cancer (stage I–IV) [18]. SNDS is the French nationwide health care insurance system database that provides claims data from several linked databases and contains information related to outpatient expenditures, hospitalizations, and deaths [19]. Combining these two databases will result in the formation of a new, enhanced data source for G/GEJ adenocarcinoma, because FREGAT data (which are generally limited to medical care provided to patients within the hospital of inclusion) will be complemented by exhaustive information about medical history, medical care, and follow-up of patients available in SNDS. This will enrich the existing disease-specific information from FREGAT with the administrative data of SNDS to fill the gaps associated with each source. Linking has been useful for prior studies leveraging the SNDS and other established European health databases [13–16,20–27]. Some studies have used direct identifiers (e.g., Central Person Register number in Denmark [20,21]), while others have used indirect identifiers (e.g., de-identified data available through the Clinical Practice Research Datalink in the United Kingdom [26,27]). SNDS and FREGAT cannot be linked directly.

In line with GUILD recommendations, we describe the record linkage process used to link patients in FREGAT with those in SNDS using a step-by-step deterministic approach [9]. The linked patient data will subsequently be evaluated to determine the value of capturing disease burden and treatment patterns for G/GEJ adenocarcinoma in France. The linkage methodology we describe will make it possible to conduct epidemiological studies of G/GEJ adenocarcinoma in France.

## Methods

### Data collection from the FREGAT and SNDS databases

The FREGAT working group was established in 2010 to coordinate the efforts of various French and French-speaking teams in the context of esophageal and gastric cancer research [18]. Data collection in the FREGAT database began in June 2014 and has expanded to include data from 42 participating centers across France, with each patient being followed over a 5-year period [18,28]. All newly diagnosed, treatment-naive, adult patients with esophageal or gastric carcinomas who received treatment at one of the participating centers were recruited into FREGAT [18]. The FREGAT database provides comprehensive data, encompassing histologic analyses of biopsied and resected tumors, blood sample analyses, epidemiologic and socioeconomic characteristics, and patient quality-of-life questionnaires.

The SNDS database was established in 2016 as an extension of the nationwide Système National d’Information Inter‐Régimes de l’Assurance Maladie database and covers the majority of the French population. Data sources within SNDS include outpatient data from Données de Consommation Inter-Régime (DCIR; a French database of billing and reimbursement records from outpatient healthcare consumption), linked hospitalization data from Programme de Médicalisation des Systèmes d’Information (PMSI; a French hospital discharge database), and causes of death from Base de Causes Médicales de Décès (BCMD; a French database of deaths and their causes) [13]. Common parameters within the SNDS data include demographics, healthcare encounters, medications (including pharmacy prescription data), medical devices, lab tests, chronic medical conditions, hospitalizations with International Classification of Diseases, Tenth Revision (ICD-10) codes for diagnoses, date and duration of hospitalization, medical procedures, diagnosis-related groups, and cost data (presented for reimbursement and actually reimbursed). Data from the SNDS database were used to identify the total population of patients with G/GEJ adenocarcinoma in France. Classification Commune des Actes Médicaux (CCAM) procedure codes and ICD-10 diagnosis codes are shown in S1 and S2 Tables, respectively.

This study was approved by the National Expertise Committee (Ethical and Scientific Committee for Research, Studies and Evaluations in the Field of Health; 5758940) and the French Personal Data Protection Agency (CNIL; 92 1441/DR 2022 088). The company responsible for data processing (RCTs, Lyon, France) complied with the French Personal Data Protection Agency criteria for SNDS access (RERC181009). When patients enrolled in FREGAT, they were informed that their anonymized data would be used for research projects and provided written consent. BECOME study information was accessible through the FREGAT database website, and patients could decline to participate by signing a withdrawal form.

SNDS data were accessed on February 2, 2023, and FREGAT data were accessed on February 21, 2023. Patient records were linked between the two databases using the definition of the variable in the FREGAT database and the corresponding variable identified in SNDS (Table 1).

**Table 1:** Common variables between FREGAT and SNDS databases used for linking.

Linkage variable(s)	FREGAT variable definition	SNDS variable(s) identified
1. Birth date	Year and month of birth	Year and month of birth
2. Sex	Sex	Sex
3. Date of consent and center ID	Date of FREGAT consent and FINESS^a^	Any identified date of in-hospital visit and corresponding FINESS^a^
4. Date of 3-year follow-up and center ID	Date of FREGAT 3-year follow-up and FINESS^a^ (recoded as missing for years >2020)	Any identified date of in-hospital visit and corresponding FINESS^a^
5. Date of death	Date of death (truncated to keep only year and month of death)	Year and month of death
6. Surgery date and center ID	First surgery date and FINESS^a^ (recoded as missing for years >2020)	Any potential surgery date (2015–2020) and corresponding FINESS^a^
7. Discharge date of the hospital admission during which surgery was performed and center ID	First surgery discharge date and FINESS^a^ (recoded as missing for years >2020)	Any potential surgery discharge date (2015–2020) and corresponding FINESS^a^
8. Type of surgery	Three binary variables for three types of surgery: gastrectomy, esophagectomy, and lymph node dissection, performed during follow-up	Three binary variables for three types of surgery: gastrectomy, esophagectomy, and lymph node dissection, performed 2015–2020
9. Chemotherapy dates	Chemotherapy start and end dates for each line for years <2021	Any date of chemotherapy session (2015–2020)
10. Radiotherapy dates	Radiotherapy start and end dates for each session for years <2021	Any date of radiotherapy session (2015–2020)
11. Endoscopic treatment date and center ID	First endoscopic treatment date and FINESS^a^ (recoded as missing for years >2020)	Any potential endoscopic treatment date (2015–2020) and corresponding FINESS^a^
12. Department of residence	Department of residence at inclusion	Departments of residence (2015–2020)

FINESS, Fichier National des Etablissements Sanitaires et Sociaux; FREGAT, FRench EsoGAstric Tumours; SNDS, Système National des Données de Santé.

^a^FINESS is an establishment identifier; only hospital establishments identified in the FREGAT database have been studied in the SNDS database.

### Inclusion and exclusion criteria

The overall French population with G/GEJ adenocarcinoma in the linked dataset had at least one insurance claim in SNDS, had a confirmed diagnosis of G/GEJ adenocarcinoma, was enrolled in FREGAT with at least two recorded visits, had an index date after January 1, 2015, and was ≥ 18 years of age at the index date. Patients with a confirmed diagnosis of GEJ squamous cell carcinoma or esophageal cancer were excluded from FREGAT.

Data were collected between January 1, 2012, and December 31, 2020. Patients in the FREGAT database who were diagnosed with G/GEJ adenocarcinoma during the selection period from January 1, 2015, to December 31, 2019, were included in the SNDS linkage. The total French population with G/GEJ adenocarcinoma, defined as patients from SNDS who had at least one insurance claim in SNDS and ICD-10 code C16 (malignant neoplasm of stomach), was included in this linkage. Patients were followed from the index date (date of histologic diagnosis or date of first metastatic relapse) to December 31, 2020, date of death, last date of coverage by an insurance plan of SNDS, or loss to follow-up. Medical history, including comorbidities, was assessed at the index date and selected from the period January 1, 2015, to December 31, 2019, as identified in FREGAT, or during the pre-index period when applicable.

### Linkage strategy

There are two primary methods of record linkage: deterministic, which relies on step-by-step rules to determine if records in two datasets belong to the same patient, and probabilistic, where records in two datasets are given scores representing likelihoods of belonging to the same individual based on the similarity of variables [29].

Because the deterministic method is the more conservative of the two, it has a higher specificity, with a reduced sensitivity for patients with missing or misclassified information; by adding multiple steps within the deterministic method with hierarchical rules, this limitation of reduced sensitivity can be mitigated [30]. Deterministic record linkage is used to link registries/databases by matching patients according to individual representative identifiers [31]. Because there are no common identifiers available between SNDS and FREGAT, patients were linked at the individual level based on matched observations for a set of variables. A step-by-step, indirect, deterministic record linkage algorithm, which matched patient records in a series of progressively less restrictive steps, was developed and implemented to match patient records from the FREGAT and SNDS databases. In this algorithm, corresponding variables in each data source were matched for the same data at an observational level based on data content, date of entry, and FINESS (Fichier National des Etablissements Sanitaires et Sociaux) code corresponding to the healthcare institution registering the observation; these matched variables were labeled as linkage variables. The goal of the linkage was to match FREGAT patients with the SNDS database while achieving the highest linkage scores possible. At steps 1–6 of the linkage process, data regarding the sex and the date of birth of each patient were required to be matched and consistent between databases to proceed to the next step of linking. Then, based on the linkage variables listed in Table 1, a total of at least four linkage variables had to match for the patient record to be considered linked.

**Fig 1:**
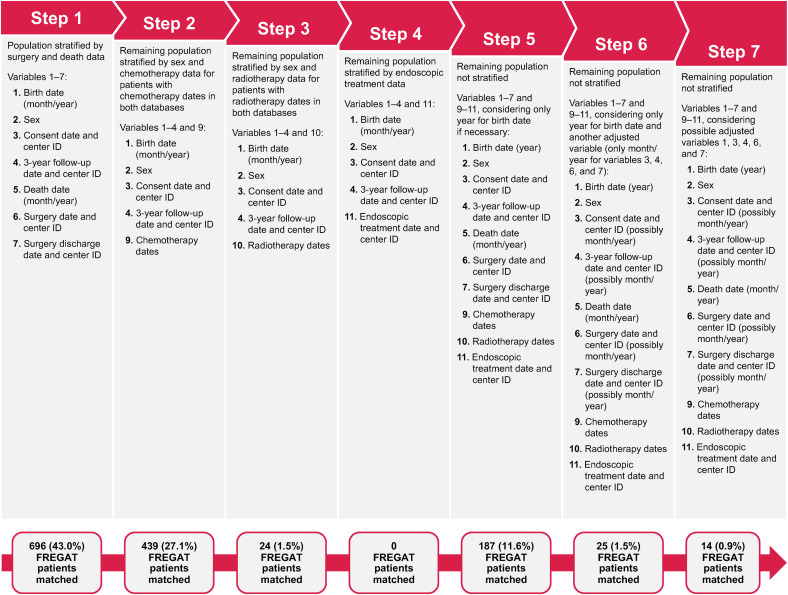
Step-by-step, indirect, deterministic record linkage algorithm for FREGAT and SNDS databases. FREGAT, FRench EsoGAstric Tumours; SNDS, Système National des Données de Santé.

Fig 1 describes the linkage process and the variables associated with each individual step. The linkage process was performed in two main parts.

First, linking was based on the stratified population (steps 1–4, with stratification based on data related to surgery and death [step 1], sex and chemotherapy [step 2], sex and radiotherapy [step 3], and endoscopic treatment [step 4]) to create smaller groups to enhance computational speed, and second, linking was conducted without any stratification of the population (steps 5–7). The first step of linkage matched patient records based on linkage variables 1–7. Patients who were not initially linked underwent two substeps that used adjusted linkage variables, first with the date of surgery in the format of month/year and then using the date of surgery discharge in the format of month/year. Patients who could not be linked across databases based on linkage variables 1–7 were matched based on linkage variables 1–4 and 9 (chemotherapy dates). Next, patients were matched based on linkage variables 1–4 and 10 (radiotherapy dates). In the fourth linkage step, patients were matched using linkage variables 1–4 and 11 (endoscopic treatment date and FINESS code). Patients in the FREGAT database who remained unlinked after the first part of the linkage (steps 1–4) were subsequently matched without any population stratification using linkage variables 1–7 and 9–11.

In the second part of the linkage (steps 5–7), the SNDS population defined as “all patients with at least one in-hospital visit” was expanded to include all patients with an in-hospital visit or stay; this was done because of the low-linking results for the variables of consent at 3-year follow-up and inconsistent provision of patient consent during the in-hospital visit (e.g., 3-year follow-up visit). In the fifth step, patients were matched based on linkage variables 1–7 and 9–11 considering only year for linkage variable 1 (date of birth), if necessary. In the sixth step of linkage, all linkage variables in step 5 were used for matching with an adjustment for only year for linkage variable 1 and another adjusted variable (only month/year for variables 3, 4, 6, and 7). In the seventh and final step of linkage, variables 1–7 and 9–11 were used for matching, including adjustments for variables 1, 3, 4, 6, and 7.

In part 1 of the linkage process, patients were required to be matched on at least four variables to be considered linked; however, in steps 2 and 3, patients were allowed to be linked if they had three matching variables with at least two matching dates. In part 2 of the linkage process, patients in steps 5 and 6 were required to be matched on at least four variables or four variables including one adjusted variable. The final step of this linkage process required patients to be matched on at least six variables.

### Statistical analysis

Descriptive and inferential statistics (i.e., chi-square, Fisher exact, and Student’s *t*-test) were used to assess the validity of the linkage process by comparing characteristics of the FREGAT non-linked and FREGAT-SNDS linked patient populations. Two-sided tests were performed using a 5% significance level. Continuous variables were summarized as number of observations (n), mean and standard deviation, median, first and third quartiles, and minimum and maximum values. Categorical variables were summarized as numbers and percentages of patients. No replacement of missing values was performed except for day of birth. When the day of birth was missing in the SNDS and FREGAT data (i.e., only month and year of birth were provided), the imputed value for the day was 01. Patients who were lost to follow-up were censored at the date of loss to follow-up.

### Quality control

In FREGAT, a data manager and a team of clinical research associates were assigned to check data quality; quality control tests were regularly performed on the electronic case report form. In SNDS, data management and analyses were performed by RCTs, a French contract research organization, in accordance with the SNDS good practice code. Data from SNDS were maintained by French National Health Insurance (Caisse Nationale de l’Assurance Maladie; CNAM) to ensure accuracy, consistency, and reproducibility.

## Results

Among the 1617 patients in the FREGAT registry who were included in the initial dataset extraction, 1385 (85.7%) were successfully linked to the SNDS dataset and 232 (14.3%) were not. Most patients were linked following the completion of part 1 (steps 1–4) of the linkage process (Table 2 and Fig 1). In step 1, 43.0% of patients from the FREGAT dataset were linked to SNDS. By linking on at least two dates of chemotherapy administration, an additional 27.1% of FREGAT patients were matched with a unique SNDS patient record, resulting in a steep increase in the cumulative percentage of patients linked. Linking based on dates of radiotherapy administration (1.5%) and endoscopic treatment (0%) resulted in few to no new linkages with the SNDS database. In part 2 (steps 5–7) of the linkage process, 14.0% of patients in FREGAT were matched without any population stratification and linked to the SNDS dataset, with most of the linkage being achieved at step 5 (11.6%).

**Table 2:** Summary of process linkage steps between FREGAT and SNDS datasets.

Step	Base linkage variables	Step-specific linkage variables	Linkage rules	Number of FREGAT patients linked (of N = 1617)	Cumulative percentage of FREGAT patients linked
Part 1: Matching on stratified population					
1	1-2	3-4-5-6-7	Score ≥4	681	42.1%
1.A	1-2	3-4-5-6^a^-7	Score ≥4	5	42.4%
1.B	1-2	3-4-5-6-7^a^	Score ≥4	10	43.0%
2	1-2	3-4-9	Score ≥4 or 3 with at least two dates	439	70.2%
3	1-2	3-4-10	Score ≥4 or 3 with at least two dates	24	71.7%
4	1-2	3-4-11	Score ≥4	0	—
Part 2: Matching without any stratification of population					
5	1^a^-2	3-4-5-6-7-9-10-11	Score ≥4	115	78.8%
			Score ≥4 (including 1 adjusted variable^a^)	72	83.2%
6	1^a^-2	3-3^a^-4-4^a^-5-6-6^a^-7-7^a^-9-10-11	Score ≥4	14	84.1%
			Score ≥4 (including 1 adjusted variable^a^)	11	84.8%
7	NA	1^a^-2-3-3^a^-4-4^a^-5-6-6^a^-7-7^a^-9-10-11	Score = 9	4	85.0%
			Score = 7	3	85.2%
			Score = 6	7	85.7%

FREGAT, FRench EsoGAstric Tumours; NA, not applicable; SNDS, Système National des Données de Santé.

^a^Adjusted variables are only MM/YYYY rather than DD/MM/YYYY, and for birth date only YYYY rather than MM/YYYY.

Chemotherapy (86.2%) and surgery (72.8%) were the treatments most frequently received for the management of G/GEJ adenocarcinoma among the linked population (Table 3). In the linked population with surgery, most patients underwent lymphadenectomy (93.7%) and gastrectomy (71.9%), while fewer patients had esophagectomy (41.4%; Table 4).

**Table 3:** Evaluation of linked and unlinked patients based on management of G/GEJ adenocarcinoma.

	FREGAT non-linked patients (n = 232)	FREGAT-SNDS linked patients (n = 1385)	*P* value^a^
**Treatment by surgery**			*P* < 0.001
** **No, n (%)	89 (38.4)	377 (27.2)	
** **Yes, n (%)	143 (61.6)	1008 (72.8)	
**Endoscopic treatment**			*P* < 0.0001
** **No, n (%)	217 (93.5)	1375 (99.3)	
** **Yes, n (%)	15 (6.5)	10 (0.7)	
**Treatment by chemotherapy**			*P* < 0.0001
** **No, n (%)	87 (37.5)	191 (13.8)	
** **Yes, n (%)	145 (62.5)	1194 (86.2)	
**Treatment by radiotherapy**			*P* = 0.292
** **No, n (%)	195 (84.1)	1124 (81.2)	
** **Yes, n (%)	37 (15.9)	261 (18.8)	

FREGAT, FRench EsoGAstric Tumours; G/GEJ, gastric/gastroesophageal junction; SNDS, Système National des Données de Santé.

^a^Statistical tests were performed to compare FREGAT non-linked patients and FREGAT-SNDS linked patients. Depending on sample size, chi-square or Fisher exact tests were performed for categorical variables.

**Table 4:** Evaluation of linked and unlinked patients based on type of surgery.

	FREGAT non-linked patients with surgery (n = 143)	FREGAT-SNDS linked patients with surgery (n = 1008)	*P* value^a^
**Gastrectomy**			*P* < 0.0001
** **No, n (%)	69 (48.3)	283 (28.1)	
** **Yes, n (%)	74 (51.7)	725 (71.9)	
**Esophagectomy**			*P* < 0.0001
** **No, n (%)	45 (31.5)	591 (58.6)	
** **Yes, n (%)	98 (68.5)	417 (41.4)	
**Lymph node dissection**			*P* = 0.313
** **No, n (%)	6 (4.2)	64 (6.3)	
** **Yes, n (%)	137 (95.8)	944 (93.7)	

FREGAT, FRench EsoGAstric Tumours; SNDS, Système National des Données de Santé.

^a^Statistical tests were performed to compare FREGAT non-linked patients and FREGAT-SNDS linked patients. Depending on sample size, chi-square or Fisher exact tests were performed for categorical variables.

The median (interquartile range) age of the linked population at diagnosis was 65.0 (55.0–73.0) years (Table 5), with most patients being male (76.4%) or having human epidermal growth factor receptor 2 (HER2)-negative (54.8%) G/GEJ adenocarcinoma. A comparison of linked and non-linked patient populations, based on treatment received, yielded *P* values <0.05 for surgical treatment (including gastrectomy and esophagectomy), endoscopy, and chemotherapy (Tables 3 and 4). Although most demographic and disease characteristics at inclusion were similar between the linked and non-linked populations, significant differences were observed for primary tumor location (*P* < 0.0001), G/GEJ adenocarcinoma status (metastatic, *P* < 0.01), HER2-negativity status (*P* < 0.01), tumor-node-metastasis (TNM) classification of malignant tumors (primary tumor status, *P* < 0.0001), and obesity (*P* = 0.010) (Tables 5 and S3). Patient characteristics in the overall FREGAT population were similar to patient characteristics of the FREGAT-SNDS linked population (Table 5).

**Table 5:** Patient characteristics in FREGAT non-linked and FREGAT-SNDS linked populations.

	Overall FREGAT dataset (N = 1617)	FREGAT non-linked patients (n = 232)	FREGAT-SNDS linked patients (n = 1385)	*P* value^a^
**Age at diagnosis, years**				
** **Mean (SD)	63.8 (12.5)	64.0 (13.3)	63.8 (12.3)	NS
** **Median (Q1, Q3)	65.0 (55.0, 73.0)	65.0 (56.0, 73.0)	65.0 (55.0, 73.0)	
**Sex, female, n (%)**	383 (23.7)	56 (24.1)	327 (23.6)	NS
**Primary tumor location**				*P* < 0.0001
** **Gastroesophageal junction, n (%)				
** **All	924 (57.1)	157 (67.7)	767 (55.4)	
** **Esophageal junction (Siewert I)	177 (10.9)	51 (22.0)	126 (9.1)	
** **Cardiac junction (Siewert II)	571 (35.3)	92 (39.7)	479 (34.6)	
** **Gastric junction under cardiac junction (Siewert III)	176 (10.9)	14 (6.0)	162 (11.7)	
** **Gastric, n (%)				
** **All	693 (42.9)	75 (32.3)	618 (44.6)	
** **Fundus	135 (8.3)	8 (3.4)	127 (9.2)	
** **Antrum	295 (18.2)	37 (15.9)	258 (18.6)	
** **Pylorus	37 (2.3)	3 (1.3)	34 (2.5)	
** **Small curvature	121 (7.5)	13 (5.6)	108 (7.8)	
** **Large curvature	46 (2.8)	6 (2.6)	40 (2.9)	
** **Pangastric	59 (3.6)	8 (3.4)	51 (3.7)	
**G/GEJ adenocarcinoma status, n (%)**				
** **Locally advanced	15 (0.9)	0	15 (1.1)	NS
** **Metastatic	655 (40.5)	73 (31.5)	582 (42.0)	*P* < 0.01
** **Unresectable	30 (1.9)	1 (0.4)	29 (2.1)	NS
**HER2-negative, n (%)**	861 (53.2)	102 (44.0)	759 (54.8)	*P* < 0.01
**TNM classification of malignant tumors at inclusion,**^**b**^ **n (%)**				
** **T0	10 (0.6)	4 (1.8)	6 (0.5)	*P* < 0.0001
** **T1	115 (7.4)	37 (17.0)	78 (5.9)	
** **T2	260 (16.8)	43 (19.7)	217 (16.3)	
** **T3	810 (52.3)	95 (43.6)	715 (53.7)	
** **T4	125 (8.1)	10 (4.6)	115 (8.6)	
** **N0	497 (31.9)	86 (38.9)	411 (30.7)	NS
** **N ≥ 1	938 (60.2)	120 (54.3)	818 (61.2)	
** **NX	123 (7.9)	15 (6.8)	108 (8.1)	
** **M0	1199 (75.6)	180 (79.3)	1019 (75.0)	NS
** **M1	347 (21.9)	42 (18.5)	305 (22.5)	
** **MX	39 (2.5)	5 (2.2)	34 (2.5)	
**Type of metastases for M1 patients, n (%)**				
** **Liver metastases	99 (28.7)	13 (31.0)	86 (28.4)	NS
** **Pulmonary metastases	24 (6.9)	5 (11.9)	19 (6.3)	NS
** **Peritoneal metastases close to the tumor	170 (49.3)	23 (54.8)	147 (48.5)	NS
** **Peritoneal metastases distant from the tumor	130 (37.7)	16 (38.1)	114 (37.6)	NS
** **Brain metastases	2 (0.6)	1 (2.4)	1 (0.3)	NS
** **Adrenal gland metastases	8 (2.3)	1 (2.4)	7 (2.3)	NS
** **Bone metastases	15 (4.3)	3 (7.1)	12 (3.9)	NS
** **Other metastases	73 (21.1)	7 (16.7)	66 (21.7)	NS
**Risk factors, n (%)**				
** **Smoking status – Yes	949 (58.7)	127 (54.7)	822 (59.4)	NS
** **Alcohol consumption – Yes	449 (27.8)	53 (22.8)	396 (28.6)	NS
** **Obesity	178 (26.1)	39 (36.8)	139 (24.2)	*P* = 0.010

FREGAT, FRench EsoGAstric Tumours; G/GEJ, gastric/gastroesophageal junction; HER2, human epidermal growth factor receptor 2; NS, nonsignificant, where *P* < 0.05 was considered significant; Q1, first quartile; Q3, third quartile; SD, standard deviation; SNDS, Système National des Données de Santé; TNM, tumor-node-metastasis.

^a^Statistical tests were performed to compare FREGAT non-linked patients and FREGAT-SNDS linked patients. Student’s *t* test was performed to compare means. Depending on sample size, chi-square or Fisher exact tests were performed for categorical variables.

^b^Inclusion is defined as the inclusion and first entry in FREGAT. This was done “at the latest on the day of treatment initiation (surgery, chemo[radio]therapy)” per FREGAT electronic case report form.

## Discussion

A linked dataset for G/GEJ adenocarcinoma in the French population may facilitate research in this area, similar to other therapeutic areas [15]. We developed an algorithm to link patients from the FREGAT and SNDS databases based on patient characteristics and treatment, using a step-by-step deterministic approach.

This step-by-step deterministic linkage approach, tailored to the French regulatory and data environments, addresses several real-world challenges, including local inconsistencies in coding, variable data quality, and limitations of pseudonymization. By transparently documenting these constraints and prioritizing deterministic linkage over probabilistic linkage, we provided a reproducible and context-sensitive framework. Beyond methodological interest, this enriched dataset offers the opportunity to evaluate treatment effectiveness, perform health economic analyses, and generate real-world evidence to support clinical and policy decision-making, among other potential analyses.

Linking the FREGAT clinico-biological database with SNDS medico-administrative data maximizes data value: It extends available information on FREGAT patients, including treatment history, comorbidities, and other events of interest; permits evaluation of the representativeness of the FREGAT database compared with the overall French population with G/GEJ cancers; and recovers information on patients who were lost to follow-up in FREGAT but have available data in SNDS, such as hospital admissions or deaths. With regard to G/GEJ adenocarcinoma research, there are many benefits. An enriched FREGAT database allows researchers and clinicians to answer epidemiologic questions related to patients with G/GEJ adenocarcinoma, including specific subgroups of patients defined based on clinical characteristics (e.g., metastatic disease or certain biomarkers). Questions regarding current disease burden, treatment patterns, clinical outcomes, and costs associated with the treatment of G/GEJ adenocarcinoma in France can be more deeply and reliably answered using consolidated data rather than analyzing the databases individually. Reviewing clinical outcomes associated with procedures and therapies for the treatment of patients with G/GEJ adenocarcinoma from FREGAT, along with the healthcare resource utilization and comorbidity data from SNDS, will allow healthcare providers to optimize care for this patient population. Finally, retrospective analysis of the linked population may inform new healthcare policies, treatment guidelines, and research to better manage patients with G/GEJ adenocarcinoma.

The step-by-step deterministic linkage approach employed here is an established approach that has been implemented in other studies [16,26,31,32]. A systematic review that analyzed research publications on data linkage with French claims data focusing on health product use and care trajectories in France found 16 studies that used indirect record linkage methodology, with nearly half (7/16) utilizing a deterministic linkage approach [17]. This linkage process has also been used for other notable databases, such as the Surveillance, Epidemiology and End Results (SEER)-Medicare linked dataset of the United States National Cancer Institute, which has demonstrated high validity and reliability [31]. Linking is endorsed by the International Council for Harmonisation of Technical Requirements for Pharmaceuticals for Human Use (ICH) as a method of mitigating issues that may arise with real-world studies (e.g., representativeness, selection bias, or missing data) and, when planned and conducted appropriately, may be used to support regulatory submissions [33,34]. Our representativeness analysis follows ICH guidance for reporting linkage. Importantly, the methodology could be used to link not only FREGAT but also other medical/oncological registries with SNDS.

In total, 86% of patients from the FREGAT database were successfully linked to the SNDS database, which was only slightly lower than reported linkage rates using direct methods (>90%), indicating that the indirect linkage methodology implemented here is almost as efficient as direct linkage in terms of linkage rate [26]. During the step-by-step linkage process, some patients had minor discrepancies in birth or death dates between the two databases. However, these patients were retained in the linked population due to high linkage scores based on strong concordance across other variables related to healthcare resource utilization. Although these patients were retained, patients with insufficient concordance of common variables (e.g., birth date, sex, and healthcare dates) identified at each step in the step-by-step linkage procedure were excluded from the linked population. Specifically, pairs with more than one potential link at any step were systematically considered linkage failures. Discrepancies in birth or death dates between the two databases among patients that were retained were likely due to isolated data entry errors, particularly in the FREGAT database. Because FREGAT is a clinico-biological database, it has the potential to have common data entry errors, such as incorrect and missing information. Inconsistencies found in clinical databases are often due to a variety of reasons, such as errors in copying the original documents into the database, errors in data interpretation, and errors in data entry [35].

There are several limitations associated with the results of this linkage. Because FREGAT and SNDS do not share the same personal identifiers for each patient, linkage errors across databases may have occurred. Given the size of this patient population, it is possible that some patients may not be linked accurately (i.e., true nonmatches may have been linked or true matches may not have been linked). This is a limitation of all record linkage processes that stems from the lack of established standards for quality evaluation. Although linkages could be manually reviewed and verified for accuracy, this process is time-consuming and requires additional legal authorizations [16]. However, considering that the aim of the linkage process is to identify unique pairs, our current methodology ensures that linked pairs are consistent between databases. Another limitation is that FREGAT primarily collects data for patients with G/GEJ adenocarcinoma from university hospitals and other specialized cancer centers [18,28]. Therefore, a potential bias exists due to the lack of inclusion of patients in FREGAT who may be receiving care at other facilities but are present in the SNDS database. In addition, this linkage approach may have a selection bias because linkage scores were based on healthcare events (e.g., chemotherapy or surgery). Patients who had higher rates of healthcare events (e.g., patients with metastatic disease or those with a higher stage of TNM classification) had a higher total score, due to a greater disease burden warranting higher levels of healthcare resource utilization, and thus these patients had a higher probability of being linked with the SNDS database compared with those without healthcare events. Patients who received little to no healthcare or those who were ineligible for treatments were underrepresented within the linked patient population and were most likely included in the FREGAT non-linked population. Lastly, the linkage approach used is specific to the FREGAT and SNDS databases; therefore, not all elements of the methodology can be applied to linkage of other databases.

In summary, using the linked FREGAT-SNDS population, further research can be done to identify the disease burden of clinically defined G/GEJ subgroups (e.g., patients with metastatic or HER2-negative disease) using data from SNDS regarding death, healthcare resource utilization, or comorbidities. The FREGAT database represents a high-quality source for epidemiologic and public health research within the scope of G/GEJ adenocarcinoma, given the high performance of linkage observed in this study. When enriched with the SNDS database, the linked data represent the most comprehensive available source of real-world data for patients with G/GEJ adenocarcinoma in France.

## Conclusion

Using a step-by-step deterministic linkage approach, we established an algorithm that enabled successful linkage of patient records from the FREGAT and SNDS databases, with a linkage rate approaching that of direct linkage. From this experience, the main parameters that would ensure optimal linkage using an indirect method are (1) data richness, ensuring sufficient variables for linkage, (2) quality of data, (3) deep knowledge of variables available from both databases, and (4) a long and iterative process to develop the most successful linkage algorithm. This algorithm may be applied in the future to capture additional data related to G/GEJ adenocarcinoma in France. By demonstrating the methodology and use of this linkage process, we hope that researchers will be aware of potential biases or limitations involved, which may improve interpretation and contextualization of results associated with linked patient data.

## Supporting information

S1 Table Classification Commune des Actes Médicaux procedure codes. (DOCX)

S2 Table International Classification of Diseases, Tenth Revision codes. (DOCX)

S3 Table Evaluation of linked and non-linked patients based on characteristics at inclusion. (DOCX)
