# Supplementary material for: Methodological approach to optimize a step-by-step deterministic linkage of SNDS data with a clinical database (FREGAT) of gastric/gastroesophageal junction adenocarcinoma in France: Pitfalls and learnings
Source: PLoS One. 2025 Nov 7;20(11):e0333667. doi: 10.1371/journal.pone.0333667 (PMC12594410; doi:10.1371/journal.pone.0333667)
Supplement: S1 Table — (DOCX) [file pone.0333667.s001.docx]

**S1 Table. Classification Commune des Actes Médicaux procedure codes.**

| **CCAM code** | **Medical procedure** | **Translation** |
| --- | --- | --- |
| **Partial gastrectomy** | | |
| HFFA003 | Gastrectomie partielle supérieure [polaire supérieure] avec rétablissement de la continuité, par laparotomie | Upper partial gastrectomy [upper pole] with restoration of continuity, by laparotomy |
| HFFC012 | Gastrectomie partielle inférieure avec anastomose gastroduodénale, par cœlioscopie | Partial lower gastrectomy with gastroduodenal anastomosis, by laparoscopy |
| HFFA002 | Gastrectomie partielle inférieure avec anastomose gastroduodénale, par laparotomie | Partial lower gastrectomy with gastroduodenal anastomosis, by laparotomy |
| HFFC002 | Gastrectomie partielle inférieure avec anastomose gastrojéjunale, par cœlioscopie | Partial lower gastrectomy with gastrojejunal anastomosis, by laparoscopy |
| HFFA006 | Gastrectomie partielle inférieure avec anastomose gastrojéjunale, par laparotomie | Partial lower gastrectomy with gastrojejunal anastomosis, by laparotomy |
| **Total gastrectomy** | | |
| HFFC017 | Gastrectomie totale avec rétablissement de la continuité, par cœlioscopie | Total gastrectomy with restoration of continuity, by laparoscopy |
| HFFA005 | Gastrectomie totale avec rétablissement de la continuité, par laparotomie | Total gastrectomy with restoration of continuity, by laparotomy |
| **Esophagectomy, without thoracotomy** | | |
| HEFA020 | Œsophagectomie totale sans rétablissement de la continuité, par cervicotomie et par laparotomie | Total esophagectomy without restoration of continuity, by cervicotomy and by laparotomy |
| HEFA004 | Œsophagectomie totale avec œsophagogastroplastie, par cervicotomie et par laparotomie | Total esophagectomy with esophagogastroplasty, by cervicotomy and by laparotomy |
| HEFA006 | Œsophagectomie totale avec œsophagocoloplastie, par cervicotomie et par laparotomie | Total esophagectomy with esophagocoloplasty, by cervicotomy and by laparotomy |
| **Esophagectomy, with thoracotomy** | | |
| HEFA022 | Œsophagectomie totale sans rétablissement de la continuité, par thoracotomie | Total esophagectomy without restoration of continuity, by thoracotomy |
| HEFA001 | Œsophagectomie avec œsophagogastroplastie, par thoracophrénotomie gauche | Esophagectomy with esophagogastroplasty, via left thoracotomy |
| HEFA013 | Œsophagectomie avec œsophagogastroplastie, par thoraco-phréno-laparotomie | Esophagectomy with esophagogastroplasty, by thoraco-phreno-laparotomy |
| HEFA003 | Œsophagectomie avec œsophagogastroplastie, par thoracotomie et par cœlioscopie | Esophagectomy with esophagogastroplasty, by thoracotomy and laparoscopy |
| HEFA012 | Œsophagectomie avec œsophagogastroplastie, par thoracotomie et par laparotomie | Esophagectomy with esophagogastroplasty, by thoracotomy and laparotomy |
| HEFA018 | Œsophagectomie avec œsophagogastroplastie, par cervicotomie, thoracotomie et cœlioscopie | Esophagectomy with esophagogastroplasty, by cervicotomy, thoracotomy, and laparoscopy |
| HEFA002 | Œsophagectomie avec œsophagogastroplastie, par cervicotomie, thoracotomie et laparotomie | Esophagectomy with esophagogastroplasty, by cervicotomy, thoracotomy and laparotomy |
| HEFA016 | Œsophagectomie avec œsophagocoloplastie, par thoraco-phréno-laparotomie | Esophagectomy with esophagocoloplasty, by thoraco-phreno-laparotomy |
| HEFA009 | Œsophagectomie avec œsophagocoloplastie, par thoracotomie et par laparotomie | Esophagectomy with esophagocoloplasty, by thoracotomy and laparotomy |
| HEFA007 | Œsophagectomie avec œsophagocoloplastie, par cervicotomie, thoracotomie et laparotomie | Esophagectomy with esophagocoloplasty, by cervicotomy, thoracotomy, and laparotomy |
| HEFA005 | Œsophagectomie avec œsophagojéjunostomie, par thoraco-phréno-laparotomie | Esophagectomy with esophagojejunostomy, by thoraco-phreno-laparotomy |
| HEFA011 | Œsophagectomie avec œsophagojéjunostomie, par thoracotomie et par laparotomie | Esophagectomy with esophagojejunostomy, by thoracotomy and laparotomy |
| **Lymph node dissection (therapeutic procedures) with similar location to FREGAT variable, for head and neck** | | |
| FCFA028 | Exérèse de nœud [ganglion] lymphatique du cou à visée thérapeutique, par cervicotomie | Excision of lymph node (ganglion) of the neck for therapeutic purposes, by cervicotomy |
| FCFA016 | Curage lymphonodal [ganglionnaire] cervical partiel unilatéral, par cervicotomie | Partial unilateral cervical lymph node dissection, by cervicotomy |
| FCFA020 | Curage lymphonodal [ganglionnaire] cervical partiel bilatéral, par cervicotomie | Partial bilateral cervical lymph node dissection, by cervicotomy |
| FCFA008 | Curage lymphonodal [ganglionnaire] cervical complet unilatéral, par cervicotomie | Complete unilateral cervical lymph node dissection, by cervicotomy |
| FCFA027 | Curage lymphonodal [ganglionnaire] cervical complet unilatéral, élargi aux muscles profonds et/ou aux nerfs du cou, à l’artère carotide externe, à la glande parotide, par cervicotomie | Complete unilateral cervical lymph node dissection, extended to the deep muscles and/or nerves of the neck, the external carotid artery, the parotid gland, by cervicotomy |
| FCFA025 | Curage lymphonodal [ganglionnaire] cervical complet unilatéral avec curage partiel controlatéral, par cervicotomie | Complete unilateral cervical lymph node dissection with contralateral partial dissection, by cervicotomy |
| FCFA013 | Curage lymphonodal [ganglionnaire] cervical complet bilatéral, par cervicotomie | Complete bilateral cervical lymph node dissection, by cervicotomy |
| FCFA005 | Curage lymphonodal [ganglionnaire] cervical complet et médiastinorécurrentiel unilatéral, par cervicotomie | Complete cervical and unilateral mediastino-recurrent lymph node dissection, by cervicotomy |
| FCFA009 | Curage lymphonodal [ganglionnaire] cervical complet et médiastinorécurrentiel bilatéral, par cervicotomie | Complete cervical and bilateral mediastino-recurrent lymph node dissection, by cervicotomy |
| FCNB001 | Sclérose de lymphangiome cervical ou cervicofacial, par injection transcutanée intralésionnelle d’agent pharmacologique | Sclerosis of cervical or cervicofacial lymphangioma, by intralesional transcutaneous injection of pharmacological agent |
| FCFA001 | Exérèse de lymphangiome cervical | Excision of cervical lymphangioma |
| FCFA003 | Exérèse de lymphangiome cervical avec extension médiastinale, par cervicotomie | Excision of cervical lymphangioma with mediastinal extension, by cervicotomy |
| FCFA026 | Exérèse de lymphangiome cervicofacial sans dissection du nerf facial | Excision of cervicofacial lymphangioma without facial nerve dissection |
| FCFA015 | Exérèse de lymphangiome cervicoparotidien | Excision of cervico-parotid lymphangioma |
| FCFA014 | Exérèse de lymphangiome cervicoparotidien avec extension buccopharyngée | Excision of cervico-parotid lymphangioma with buccopharyngeal extension |
| FCFA023 | Exérèse de lymphangiome cervicoparotidien avec extension buccopharyngée et médiastinale, par cervicotomie | Excision of cervico-parotid lymphangioma with buccopharyngeal and mediastinal extension, by cervicotomy |
| **Lymph node dissection (therapeutic procedures) with similar location to FREGAT variable, for thorax** | | |
| FCFC002 | Exérèse de nœud [ganglion] lymphatique du médiastin, par thoracoscopie ou par médiastinoscopie | Mediastinal lymph node excision, by thoracoscopy or mediastinoscopy |
| FCFA002 | Curage lymphonodal [ganglionnaire] médiastinal supérieur, par cervicotomie | Upper mediastinal lymph node dissection, by cervicotomy |
| FCFA004 | Curage lymphonodal [ganglionnaire] médiastinal unilatéral ou bilatéral, par thoracotomie | Unilateral or bilateral mediastinal lymph node dissection, by thoracotomy |
| **Lymph node dissection (therapeutic procedures) with similar location to FREGAT variable, for abdomen** | | |
| FCFC003 | Curage lymphonodal [ganglionnaire] pelvien, par cœlioscopie ou par rétropéritonéoscopie | Pelvic lymph node dissection, by laparoscopy or retroperitoneoscopy |
| FCFA006 | Curage lymphonodal [ganglionnaire] pelvien, par laparotomie | Pelvic lymph node dissection, by laparotomy |
| FCFC001 | Curage lymphonodal [ganglionnaire] iliaque, par cœlioscopie ou par rétropéritonéoscopie | Iliac lymph node dissection, by laparoscopy or retroperitoneoscopy |
| FCFA019 | Curage lymphonodal [ganglionnaire] iliaque unilatéral ou bilatéral, par laparotomie | Unilateral or bilateral iliac lymph node dissection, by laparotomy |
| FCFC005 | Curage lymphonodal [ganglionnaire] lomboaortique, par cœlioscopie ou par rétropéritonéoscopie | Lumbo-aortic lymph node dissection, by laparoscopy or retroperitoneoscopy |
| FCFA010 | Curage lymphonodal [ganglionnaire] lomboaortique, par laparotomie | Lumbo-aortic lymph node dissection, by laparotomy |
| FCFC004 | Curage lymphonodal [ganglionnaire] lomboaortique avec curage iliaque unilatéral ou bilatéral, par cœlioscopie ou par rétropéritonéoscopie | Lumbo-aortic lymph node dissection with unilateral or bilateral iliac dissection, by laparoscopy or retroperitoneoscopy |
| FCFA022 | Curage lymphonodal [ganglionnaire] lomboaortique avec curage iliaque unilatéral ou bilatéral, par laparotomie | Lumbo-aortic lymph node dissection with unilateral or bilateral iliac dissection, by laparotomy |
| **Endoscopic procedures** | | |
| HEME900 | Correction d’un reflux gastroœsophagien, par endoscopie | Correction of gastroesophageal reflux, by endoscopy |
| HENE494 | Destruction localisée de la muqueuse de l’œsophage par radiofréquence, par œso-gastro-duodénoscopie | Localized destruction of the esophageal mucosa by radiofrequency, by esophagogastroduodenoscopy |
| HENE900 | Séance de destruction d’une hétérotopie de la muqueuse de l’œsophage inférieur, par endoscopie | Endoscopic destruction of a heterotopia of the mucosa of the lower esophagus |
| HENE001 | Séance de destruction photodynamique de lésion de l’œsophage et/ou de l’estomac avec laser, par œso-gastro-duodénoscopie | Photodynamic destruction of lesions of the esophagus and/or stomach with laser, by esophagogastroduodenoscopy |
| HENE002 | Séance de destruction de lésion de l’œsophage et/ou de l’estomac avec laser, par œso-gastro-duodénoscopie | Destruction of lesions of the esophagus and/or stomach with laser, by esophagogastroduodenoscopy |
| HENE004 | Séance de destruction de lésion de l’œsophage et/ou de l’estomac sans laser, par œso-gastro-duodénoscopie | Destruction of lesions of the esophagus and/or stomach without laser, by esophagogastroduodenoscopy |
| HEFE003 | Séance de mucosectomie de l’œsophage, de l’estomac ou du duodénum, par œso-gastro-duodénoscopie | Mucosectomy of the esophagus, stomach or duodenum, by esophagogastroduodenoscopy |
| HEFE326 | Dissection sousmuqueuse de l’œsophage et/ou de l’estomac, par œso-gastro-duodénoscopie | Submucosal dissection of the esophagus and/or stomach, by esophagogastroduodenoscopy |
| HEFE001 | Exérèse d’un polype de 1cm et plus de diamètre ou de 4 polypes ou plus de l’œsophage, de l’estomac et/ou du duodénum, par œso-gastro-duodénoscopie | Removal of a polyp 1 cm or more in diameter or 4 or more polyps from the esophagus, stomach, and/or duodenum, by esophagogastroduodenoscopy |
| HEFE002 | Exérèse de 1 à 3 polypes de moins de 1cm de diamètre de l’œsophage, de l’estomac et/ou du duodénum, par œso-gastro-duodénoscopie | Removal of 1–3 polyps less than 1 cm in diameter from the esophagus, stomach, and/or duodenum, by esophagogastroduodenoscopy |
| HEGE002 | Ablation de corps étranger de l’œsophage, de l’estomac et/ou du duodénum, par œso-gastro-duodénoscopie | Removal of foreign bodies from the esophagus, stomach, and/or duodenum, by esophagogastroduodenoscopy |
| HFLE001 | Pose d’une sonde gastrique, duodénale ou jéjunale, par œso-gastro-duodénoscopie | Placement of a gastric, duodenal or jejunal tube, by esophagogastroduodenoscopy |
| HFLH001 | Pose d’une sonde gastrique, duodénale ou jéjunale, avec guidage radiologique | Placement of a gastric, duodenal or jejunal tube, with radiological guidance |
| HEAE001 | Dilatation rétrograde de l’œsophage, par endoscopie rigide | Retrograde dilation of the esophagus, by rigid endoscopy |
| HEAE002 | Dilatation antérograde de l’œsophage, par endoscopie rigide | Antegrade dilation of the esophagus, by rigid endoscopy |
| HEAE003 | Dilatation antérograde de l’œsophage, par fibroscopie | Antegrade dilation of the esophagus, by fibroscopy |
| HEAH001 | Dilatation de l’œsophage, avec guidage radiologique | Dilation of the esophagus, with radiological guidance |
| HEGE003 | Ablation d’une endoprothèse de l’œsophage, par endoscopie | Removal of an esophageal stent, by endoscopy |
| HEKE001 | Changement d’une endoprothèse de l’œsophage, par endoscopie | Changing an esophageal stent, by endoscopy |
| HELE002 | Pose d’une endoprothèse de l’œsophage, par endoscopie | Endoscopic insertion of an esophageal stent |
| HELH001 | Pose d’une endoprothèse de l’œsophage, avec guidage radiologique | Placement of an esophageal stent, with radiological guidance |

Translations are not validated and are provided for informational purposes only.

CCAM, Classification Commune des Actes Médicaux; FREGAT, FRench EsoGAstric Tumours.
