# Supplementary material for: Methodological approach to optimize a step-by-step deterministic linkage of SNDS data with a clinical database (FREGAT) of gastric/gastroesophageal junction adenocarcinoma in France: Pitfalls and learnings
Source: PLoS One. 2025 Nov 7;20(11):e0333667. doi: 10.1371/journal.pone.0333667 (PMC12594410; doi:10.1371/journal.pone.0333667)
Supplement: S2 Table — (DOCX) [file pone.0333667.s002.docx]

**S2 Table. International Classification of Diseases, Tenth Revision codes.**

| **ICD-10 codes** | **Description** | **Further Description** |
| --- | --- | --- |
| C16 | Malignant neoplasm of stomach |  |
| C16.0 | Malignant neoplasm of cardia | Malignant neoplasm of cardiac orifice  Malignant neoplasm of cardio-esophageal junction  Malignant neoplasm of esophagus and stomach  Malignant neoplasm of gastro-esophageal junction |
| C16.1 | Malignant neoplasm of fundus of stomach |  |
| C16.2 | Malignant neoplasm of body of stomach |  |
| C16.3 | Malignant neoplasm of pyloric antrum | Malignant neoplasm of gastric antrum |
| C16.4 | Malignant neoplasm of pylorus | Malignant neoplasm of prepylorus  Malignant neoplasm of pyloric canal |
| C16.5 | Malignant neoplasm of lesser curvature of stomach, unspecified |  |
| C16.6 | Malignant neoplasm of greater curvature of stomach, unspecified |  |
| C16.8 | Malignant neoplasm of overlapping sites of stomach |  |
| C16.9 | Malignant neoplasm of stomach, unspecified | Gastric cancer NOS |
| Z51.0 | Encounter for antineoplastic radiation therapy |  |
| Z51.1 | Encounter for antineoplastic chemotherapy and immunotherapy |  |

ICD-10, International Classification of Diseases, Tenth Revision.
