# Supplementary material for: Methodological approach to optimize a step-by-step deterministic linkage of SNDS data with a clinical database (FREGAT) of gastric/gastroesophageal junction adenocarcinoma in France: Pitfalls and learnings
Source: PLoS One. 2025 Nov 7;20(11):e0333667. doi: 10.1371/journal.pone.0333667 (PMC12594410; doi:10.1371/journal.pone.0333667)
Supplement: S3 Table — (DOCX) [file pone.0333667.s003.docx]

**S3 Table. Evaluation of linked and non-linked patients based on characteristics at inclusion.**

|  | **FREGAT non-linked patients (n=232)** | **FREGAT-SNDS linked patients (n=1385)** | ***P* value^a^** |
| --- | --- | --- | --- |
| **Age at diagnosis** |  |  |  |
| Non-missing | 231 | 1382 |  |
| Mean (SD), years | 64.0 (13.3) | 63.8 (12.3) | *P*=0.860 |
| Median, years | 65.0 | 65.0 |  |
| Q1; Q3, years | 56.0; 73.0 | 55.0; 73.0 |  |
| Min; max, years | 19.0; 93.0 | 22.0; 95.0 |  |
| Missing | 1 | 3 |  |
| **Calendar year of diagnosis** |  |  | *P*=0.596 |
| Non-missing | 231 | 1382 |  |
| 2014, n (%) | 3 (1.3) | 27 (2.0) |  |
| 2015, n (%) | 35 (15.2) | 229 (16.6) |  |
| 2016, n (%) | 51 (22.1) | 339 (24.5) |  |
| 2017, n (%) | 57 (24.7) | 296 (21.4) |  |
| 2018, n (%) | 50 (21.6) | 279 (20.2) |  |
| 2019, n (%) | 34 (14.7) | 211 (15.3) |  |
| 2020, n (%) | 1 (0.4) | 1 (0.1) |  |
| Missing | 1 | 3 |  |
| **Sex** |  |  | *P*=0.861 |
| Non-missing | 232 | 1385 |  |
| Male, n (%) | 176 (75.9) | 1058 (76.4) |  |
| Female, n (%) | 56 (24.1) | 327 (23.6) |  |
| Missing | 0 | 0 |  |
| **Smoking status** |  |  | *P*=0.183 |
| Non-missing | 232 | 1384 |  |
| No, n (%) | 105 (45.3) | 562 (40.6) |  |
| Yes, n (%) | 127 (54.7) | 822 (59.4) |  |
| Missing | 0 | 1 |  |
| **Alcohol consumption** |  |  | *P*=0.069 |
| Non-missing | 232 | 1383 |  |
| No, n (%) | 179 (77.2) | 987 (71.4) |  |
| Yes, n (%) | 53 (22.8) | 396 (28.6) |  |
| Missing | 0 | 2 |  |
| **Primary tumor location** |  |  | *P*<0.0001 |
| Non-missing | 232 | 1385 |  |
| GEJ: esophageal junction (Siewert I), n (%) | 51 (22.0) | 126 (9.1) |  |
| GEJ: cardiac junction (Siewert II), n (%) | 92 (39.7) | 479 (34.6) |  |
| GEJ: gastric junction under cardiac junction (Siewert III), n (%) | 14 (6.0) | 162 (11.7) |  |
| Gastric: fundus, n (%) | 8 (3.4) | 127 (9.2) |  |
| Gastric: antrum, n (%) | 37 (15.9) | 258 (18.6) |  |
| Gastric: pylorus, n (%) | 3 (1.3) | 34 (2.5) |  |
| Gastric: small curvature, n (%) | 13 (5.6) | 108 (7.8) |  |
| Gastric: large curvature, n (%) | 6 (2.6) | 40 (2.9) |  |
| Gastric: pangastric, n (%) | 8 (3.4) | 51 (3.7) |  |
| Missing | 0 | 0 |  |
| **Histologic type adenocarcinoma** |  |  | NA |
| Non-missing | 232 | 1385 |  |
| Yes, n (%) | 232 (100.0) | 1385 (100.0) |  |
| Missing | 0 | 0 |  |
| **Locally advanced G/GEJ adenocarcinoma** |  |  | *P*=0.148 |
| Non-missing | 232 | 1385 |  |
| No, n (%) | 232 (100.0) | 1370 (98.9) |  |
| Yes, n (%) | 0 | 15 (1.1) |  |
| Missing | 0 | 0 |  |
| **Metastatic G/GEJ adenocarcinoma** |  |  | *P*=0.002 |
| Non-missing | 232 | 1385 |  |
| No, n (%) | 159 (68.5) | 803 (58.0) |  |
| Yes, n (%) | 73 (31.5) | 582 (42.0) |  |
| Missing | 0 | 0 |  |
| **Unresectable G/GEJ adenocarcinoma** |  |  | *P*=0.111 |
| Non-missing | 232 | 1385 |  |
| No, n (%) | 231 (99.6) | 1356 (97.9) |  |
| Yes, n (%) | 1 (0.4) | 29 (2.1) |  |
| Missing | 0 | 0 |  |
| **Histologic type at diagnosis** |  |  | *P*=0.388 |
| Non-missing | 232 | 1385 |  |
| Adenocarcinoma, n (%) | 219 (94.4) | 1325 (95.7) |  |
| Other, n (%) | 13 (5.6) | 60 (4.3) |  |
| Missing | 0 | 0 |  |
| **HER2-negative** |  |  | *P*=0.002 |
| Non-missing | 232 | 1385 |  |
| No, n (%) | 130 (56.0) | 626 (45.2) |  |
| Yes, n (%) | 102 (44.0) | 759 (54.8) |  |
| Missing | 0 | 0 |  |
| **TNM classification, T** |  |  | *P*<0.0001 |
| Non-missing | 218 | 1332 |  |
| T0, n (%) | 4 (1.8) | 6 (0.5) |  |
| T1, n (%) | 37 (17.0) | 78 (5.9) |  |
| T2, n (%) | 43 (19.7) | 217 (16.3) |  |
| T3, n (%) | 95 (43.6) | 715 (53.7) |  |
| T4, n (%) | 10 (4.6) | 115 (8.6) |  |
| Other, n (%) | 29 (13.3) | 201 (15.1) |  |
| Missing | 14 | 53 |  |
| **TNM classification, N** |  |  | *P*=0.053 |
| Non-missing | 221 | 1337 |  |
| N0, n (%) | 86 (38.9) | 411 (30.7) |  |
| N ≥1, n (%) | 120 (54.3) | 818 (61.2) |  |
| NX, n (%) | 15 (6.8) | 108 (8.1) |  |
| Missing | 11 | 48 |  |
| **TNM classification, M** |  |  | *P*=0.381 |
| Non-missing | 227 | 1358 |  |
| M0, n (%) | 180 (79.3) | 1019 (75.0) |  |
| M1, n (%) | 42 (18.5) | 305 (22.5) |  |
| MX, n (%) | 5 (2.2) | 34 (2.5) |  |
| Missing | 5 | 27 |  |
| **Among patients CM1, type of metastases** |  |  | *P*=0.730 |
| Liver metastases |  |  |  |
| Non-missing | 42 | 303 |  |
| No, n (%) | 29 (69.0) | 217 (71.6) |  |
| Yes, n (%) | 13 (31.0) | 86 (28.4) |  |
| Missing | 0 | 2 |  |
| Pulmonary metastases |  |  | *P*=0.191 |
| Non-missing | 42 | 304 |  |
| No, n (%) | 37 (88.1) | 285 (93.8) |  |
| Yes, n (%) | 5 (11.9) | 19 (6.3) |  |
| Missing | 0 | 1 |  |
| Peritoneal metastases close to the tumor |  |  | *P*=0.448 |
| Non-missing | 42 | 303 |  |
| No, n (%) | 19 (45.2) | 156 (51.5) |  |
| Yes, n (%) | 23 (54.8) | 147 (48.5) |  |
| Missing | 0 | 2 |  |
| Peritoneal metastases distant from the tumor |  |  | *P*=0.953 |
| Non-missing | 42 | 303 |  |
| No, n (%) | 26 (61.9) | 189 (62.4) |  |
| Yes, n (%) | 16 (38.1) | 114 (37.6) |  |
| Missing | 0 | 2 |  |
| Brain metastases |  |  | *P*=0.228 |
| Non-missing | 42 | 304 |  |
| No, n (%) | 41 (97.6) | 303 (99.7) |  |
| Yes, n (%) | 1 (2.4) | 1 (0.3) |  |
| Missing | 0 | 1 |  |
| Adrenal gland metastases |  |  | *P*=1.000 |
| Non-missing | 42 | 304 |  |
| No, n (%) | 41 (97.6) | 297 (97.7) |  |
| Yes, n (%) | 1 (2.4) | 7 (2.3) |  |
| Missing | 0 | 1 |  |
| Bone metastases |  |  | *P*=0.407 |
| Non-missing | 42 | 304 |  |
| No, n (%) | 39 (92.9) | 292 (96.1) |  |
| Yes, n (%) | 3 (7.1) | 12 (3.9) |  |
| Missing | 0 | 1 |  |
| Other metastases |  |  | *P*=0.453 |
| Non-missing | 42 | 304 |  |
| No, n (%) | 35 (83.3) | 238 (78.3) |  |
| Yes, n (%) | 7 (16.7) | 66 (21.7) |  |
| Missing | 0 | 1 |  |
| **History of gastroesophageal reflux** |  |  | *P*=0.074 |
| Non-missing | 231 | 1384 |  |
| No, n (%) | 153 (66.2) | 1017 (73.5) |  |
| Yes, n (%) | 73 (31.6) | 344 (24.9) |  |
| Not known, n (%) | 5 (2.2) | 23 (1.7) |  |
| Missing | 1 | 1 |  |
| **Other risk factor** |  |  | *P*=0.200 |
| Non-missing | 230 | 1383 |  |
| No, n (%) | 124 (53.9) | 808 (58.4) |  |
| Yes, n (%) | 106 (46.1) | 575 (41.6) |  |
| Missing | 2 | 2 |  |
| **Among patients with other risk factor, type of risk factor** |  |  |  |
| HPV infection |  |  | *P*=0.891 |
| Non-missing | 106 | 575 |  |
| No, n (%) | 100 (94.3) | 541 (94.1) |  |
| Yes, n (%) | 0 | 3 (0.5) |  |
| Not known, n (%) | 6 (5.7) | 31 (5.4) |  |
| Missing | 0 | 0 |  |
| Esophagitis |  |  | *P*=0.463 |
| Non-missing | 104 | 575 |  |
| No, n (%) | 94 (90.4) | 532 (92.5) |  |
| Yes, n (%) | 6 (5.8) | 32 (5.6) |  |
| Not known, n (%) | 4 (3.8) | 11 (1.9) |  |
| Missing | 2 | 0 |  |
| Chronic atrophic gastritis |  |  | *P*=0.441 |
| Non-missing | 106 | 575 |  |
| No, n (%) | 91 (85.8) | 505 (87.8) |  |
| Yes, n (%) | 12 (11.3) | 63 (11.0) |  |
| Not known, n (%) | 3 (2.8) | 7 (1.2) |  |
| Missing | 0 | 0 |  |
| Gastric ulcer |  |  | *P*=0.274 |
| Non-missing | 105 | 575 |  |
| No, n (%) | 85 (81.0) | 433 (75.3) |  |
| Yes, n (%) | 18 (17.1) | 136 (23.7) |  |
| Not known, n (%) | 2 (1.9) | 6 (1.0) |  |
| Missing | 1 | 0 |  |
| Chronic hypertrophic gastritis |  |  | *P*=0.831 |
| Non-missing | 104 | 574 |  |
| No, n (%) | 97 (93.3) | 539 (93.9) |  |
| Yes, n (%) | 4 (3.8) | 23 (4.0) |  |
| Not known, n (%) | 3 (2.9) | 12 (2.1) |  |
| Missing | 2 | 1 |  |
| History of partial gastrectomy |  |  | *P*=0.291 |
| Non-missing | 106 | 575 |  |
| No, n (%) | 102 (96.2) | 551 (95.8) |  |
| Yes, n (%) | 2 (1.9) | 20 (3.5) |  |
| Not known, n (%) | 2 (1.9) | 4 (0.7) |  |
| Missing | 0 | 0 |  |
| *CDH1* mutation |  |  | *P*=0.304 |
| Non-missing | 106 | 575 |  |
| No, n (%) | 95 (89.6) | 531 (92.3) |  |
| Yes, n (%) | 1 (0.9) | 2 (0.3) |  |
| Not known, n (%) | 10 (9.4) | 42 (7.3) |  |
| Missing | 0 | 0 |  |
| HNPCC syndrome |  |  | *P*=0.601 |
| Non-missing | 106 | 575 |  |
| No, n (%) | 102 (96.2) | 557 (96.9) |  |
| Yes, n (%) | 1 (0.9) | 2 (0.3) |  |
| Not known, n (%) | 3 (2.8) | 16 (2.8) |  |
| Missing | 0 | 0 |  |
| Familial polyadenomatosis |  |  | *P*=0.533 |
| Non-missing | 106 | 575 |  |
| No, n (%) | 96 (90.6) | 531 (92.3) |  |
| Not known, n (%) | 10 (9.4) | 44 (7.7) |  |
| Missing | 0 | 0 |  |
| Peutz-Jeghers syndrome |  |  | *P*=1.000 |
| Non-missing | 106 | 575 |  |
| No, n (%) | 104 (98.1) | 565 (98.3) |  |
| Not known, n (%) | 2 (1.9) | 10 (1.7) |  |
| Missing | 0 | 0 |  |
| Obesity |  |  | *P*=0.010 |
| Non-missing | 106 | 575 |  |
| No, n (%) | 65 (61.3) | 432 (75.1) |  |
| Yes, n (%) | 39 (36.8) | 139 (24.2) |  |
| Not known, n (%) | 2 (1.9) | 4 (0.7) |  |
| Missing | 0 | 0 |  |

FREGAT, FRench EsoGAstric Tumours; GEJ, gastroesophageal junction; G/GEJ, gastric/gastroesophageal junction; HER2, human epidermal growth factor receptor 2; HNPCC, hereditary nonpolyposis colorectal cancer (Lynch syndrome); HPV, human papillomavirus; M, status of distant metastasis; NA, not applicable; N, status of lymph node involvement; Q1, first quartile; Q3, third quartile; SD, standard deviation; SNDS, Système National des Données de Santé; T, status of primary tumor; TNM, tumor-node-metastasis.

^a^Statistical tests were performed to compare FREGAT non-linked patients and FREGAT-SNDS linked patients. Student’s *t* test was performed to compare means. Depending on sample size, chi-square or Fisher exact tests were performed for categorical variables.
